# The impact of lifecourse socio-economic position and individual social mobility on breast cancer risk

**DOI:** 10.1186/s12885-020-07648-w

**Published:** 2020-11-23

**Authors:** Eloïse Berger, Noële Maitre, Francesca Romana Mancini, Laura Baglietto, Vittorio Perduca, Hélène Colineaux, Sabina Sieri, Salvatore Panico, Carlotta Sacerdote, Rosario Tumino, Paolo Vineis, Marie-Christine Boutron-Ruault, Gianluca Severi, Raphaële Castagné, Cyrille Delpierre

**Affiliations:** 1 UMR LEASP, Université de Toulouse III, UPS, Inserm, Toulouse, France; 2 CESP, Faculté de Médecine, Université Paris-Saclay, UVSQ, INSERM, Villejuif, France; 3 Gustave Roussy, F-94805 Villejuif, France; 4 Department of Clinical and Experimental Medicine, University of Pisa, Pisa, Italy; 5 Université de Paris, CNRS, MAP5 UMR 8145, F-75006 Paris, France; 6 Epidemiology Department, Toulouse Teaching Hospital, Toulouse, France; 7 Epidemiology and Prevention Unit, Fondazione IRCCS Istituto Nazionale dei Tumori, Milan, Italy; 8 Department of Clinical Medicine and Surgery, University of Naples Federico II, Naples, Italy; 9 Unit of Cancer Epidemiology, Città della Salute e della Scienza University-Hospital and Center for Cancer Prevention (CPO), Turin, Italy; 10 Cancer Registry and Histopathology Department, Provicial Health Authority (ASP) Ragusa, Ragusa, Italy; 11 Department of Epidemiology and Biostatistics, Imperial College London, MRC-PHE Centre for Environment and Health, School of Public Health, London, UK; 12 Italian Institute for Genomic Medicine, Torino, Italy; 13 Department of Statistics, Computer Science and Applications “G. Parenti” (DISIA), University of Florence, Florence, Italy

**Keywords:** Lifecourse socio-economic position, Social mobility, Breast cancer, Prospective cohorts

## Abstract

**Background:**

Women with an advantaged socioeconomic position (SEP) have a higher risk of developing breast cancer (BC). The reasons for this association do not seem to be limited to reproductive factors and remain to be understood. We aimed to investigate the impact of lifecourse SEP from childhood and social mobility on the risk of BC considering a broad set of potential mediators.

**Methods:**

We used a discovery-replication strategy in two European prospective cohorts, E3N (*N* = 83,436) and EPIC-Italy (*N* = 20,530). In E3N, 7877 women were diagnosed with BC during a median 24.4 years of follow-up, while in EPIC-Italy, 893 BC cases were diagnosed within 15.1 years. Hazard ratios (HR) were estimated using Cox proportional hazard models on imputed data.

**Results:**

In E3N, women with higher education had a higher risk of BC (HR [95%CI] = 1.21 [1.12, 1.30]). This association was attenuated by adjusting for reproductive factors, in particular age at first childbirth (HR[95%CI] = 1.13 [1.04, 1.22]). Health behaviours, anthropometric variables, and BC screening had a weaker effect on the association. Women who remained in a stable advantaged SEP had a higher risk of BC (HR [95%CI] = 1.24 [1.07; 1.43]) attenuated after adjustment for potential mediators (HR [95%CI] = 1.13 [0.98; 1.31]). These results were replicated in EPIC-Italy.

**Conclusions:**

These results confirm the important role of reproductive factors in the social gradient in BC risk, which does not appear to be fully explained by the large set of potential mediators, including cancer screening, suggesting that further research is needed to identify additional mechanisms.

**Supplementary Information:**

The online version contains supplementary material available at 10.1186/s12885-020-07648-w.

## Background

Women with an advantaged socioeconomic position (SEP) have a higher risk of developing breast cancer (BC) compared to their disadvantaged counterparts [1, 2].

The social inequalities in BC risk could be partly explained by socially stratified distribution of known BC risk factors [3–7]. In particular age at first childbirth and parity seem to explain a large part of the association between SEP and the risk of BC [8–15]. However, an independent association between SEP and risk of BC has also been observed after controlling for those reproductive factors [8, 9, 11, 14].

Altogether these studies highlight the importance of reproductive factors in the social gradient of BC but also suggest that other pathways and mechanisms are involved and remain to be characterized. The few studies that have additionally considered health behaviours or anthropometric factors have shown that women with higher early life SEP had a higher risk of BC, partly mediated by age at first childbirth and the number of children but not by health behaviours [9]. In addition, several studies suggest that screening could act as a mediator in the association between SEP and the risk of BC [8, 12, 16, 17].

Most available studies so far have mainly focused on either young adulthood or late adulthood SEP [8, 10–13, 15–18], and few have examined SEP at different time points within a lifecourse framework [9, 14, 17, 19]. Studying the respective and combined effect of childhood and adult SEP is needed because they may reveal different mechanisms involved in the social gradient of BC incidence.

One of the major limitations of previous studies is the lack of simultaneous consideration of all potential mediators identified over the last two decades. It remains unclear, whether all those mediators influence the association between SEP and the risk of BC, and which ones are the main drivers of the association. We therefore aimed to investigate the potential and mutual impact of a large set of reproductive factors, anthropometric characteristics, and health behaviours, within a lifecourse framework. The purpose was to simultaneously evaluate all potential mediators, and to establish to which extent they can explain the social inequalities associated with breast cancer risk.

More specifically, we first assessed the relationship between SEP, from childhood to adulthood, and the future risk of BC in the E3N cohort. We further examined the impact of health behaviours, anthropometric characteristics, reproductive factors, family history of hormone-related cancer, and BC screening on these relationships. Third, we investigated the lifecourse influence of each SEP by sequentially controlling for time-ordered SEP, and investigated the impact of social mobility on BC risk. Finally, to assess the robustness and test the generalisability of our results, we conducted an independent replication study in the EPIC-Italy cohort.

## Methods

### Study populations

E3N and EPIC-Italy are two cohorts included in the European Prospective Investigation into Cancer and Nutrition (EPIC) study and have been described in detail elsewhere [20, 21]. Additional information is available in Additional file 1.

Briefly, E3N includes 98,995 women aged 38–66 and insured by the Mutuelle Générale de l’Education Nationale (MGEN), a national health insurance plan that primarily covers teachers. At inclusion, participants provided a written informed consent for the study and filled in a questionnaire that collected information about anthropometric measures, lifestyle / behaviours, SEP, and health. Self-administered questionnaires have been subsequently sent every 2–3 years since 1990. The June 1993 questionnaire (Q3) included a detailed diet history questionnaire.

EPIC-Italy represents a total of 34,152 volunteers aged 30–75 years at inclusion and recruited from four centers in Italy in 1993–1998. At inclusion, participants have filled in a questionnaire that collected information about anthropometric measurements, lifestyle / behaviours, SEP, and health.

### Lifecourse SEP

We selected lifecourse SEP among variables available in both cohorts. SEP was measured at three time points from childhood to adulthood based on self-reported information and classified as described elsewhere [22], with some adaptation as detailed in Additional file 2.

Self-reported father’s occupation was used as a proxy for childhood SEP. E3N women were born 1925–1950 and grew up in the 1940–60s, when the father’s occupation could be considered a good indicator of the household’s socio-economic conditions. We applied 3 E-SEC categories: less advantaged occupations [lower clerical, services, and sales workers; skilled workers; semi and unskilled workers (Class 7–9 ESEC)]; medium occupations [small employers and self-employed; farmers; lower supervisors and technicians (Class 4, 5, and 6 ESEC)] and more advantaged occupations [higher professionals and managers, lower professionals and managers; higher clerical, services and sales workers (Class 1–3 ESEC)].

SEP in young adulthood was measured using the participant’s education categorised in 3 groups: low level [primary or lower secondary school], middle level [higher secondary school], and high level attainment [tertiary education].

Adulthood SEP was measured by the women’s own occupation, following the same categorisation as for the father’s occupation.

In EPIC-Italy, there was a large proportion of housewives, thus we used the highest household occupation to define adulthood SEP.

### Follow up and outcomes

In E3N, self-reported BC diagnosis was confirmed through pathological reports. Women were followed from inclusion (Q1, 1990) to the date of BC diagnosis last filled in questionnaire, or end of the study (2014), whichever occurred first.

In EPIC-Italy, cancer cases were identified through automated linkage to cancer and mortality registries, or through active follow-up, and confirmed with histological reports or hospital discharge systems. Follow up time started at inclusion (1993–1998) to the last follow up: December 2010 for women from Naples, Turin, and Ragusa, and to December 2009 for women from Varese.

### Covariates

Description of all covariates is provided in Additional file 3. The following factors have been considered as intermediate variables that could mediate the relationship between SEP and the risk of BC, and categorised in two blocks: i) Health behaviours and anthropometric characteristics including *alcohol consumption*; *smoking status*; *physical activity*; *Western diet pattern*; *weight;* and *height*; ii) reproductive factors including *menopausal status*; u*se of menopausal hormone therapy (MHT)*; *age at first childbirth*; *breastfeeding*; *reproductive lifespan*. We also included *family history of a hormone-related cancer (ovarian and breast);* and *BC screening*.

**Fig. 1:**
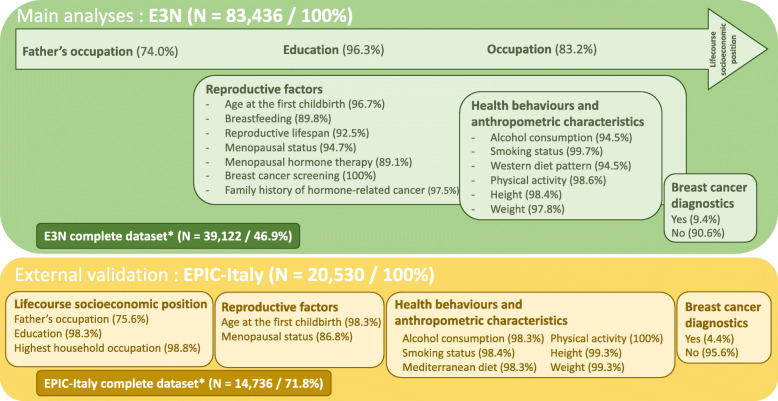
Overview of selected data among both cohorts and proportion of available data from the study population % represents proportion of women with available data for each covariate in the population before imputation; * Sub-dataset including women with all available data among selected covariates in each cohort

Description of all variables used is provided in Fig. 1.

In EPIC-Italy, available data were not exactly the same as those available in E3N. We selected and considered i) health behaviours and anthropometric characteristics: *alcohol consumption, smoking status, physical activity* (adapted from [23])*, Mediterranean diet* [24]*, height, and weight;* and ii) reproductive factors: *age at first childbirth, and menopausal status.*

### Statistical analyses in E3N

#### Descriptive analyses

Baseline characteristics of the population were compared by BC status and for each SEP indicator. Chi-squared test or Fisher’s exact test for categorical variables, and T-test or Wilcoxon rank test for continuous variables were used to estimate bivariate associations with SEP and log rank for the association with the risk of BC. Only those associated with both SEP and BC (*p*-value < 0.2) were considered in the multivariate and lifecourse analyses, except for smoking which was systematically included since it has been found associated with both SEP and BC in the literature.

#### Multivariate analyses

Cox proportional hazard models were used to investigate the relationship between SEP and BC risk. We defined a first model only adjusted for age (Model1). From Model 1, we adjusted for each potential mediator from each block of variables and also by block of variables as defined above. Finally, we defined a fully adjusted model including both blocks of variables. For each of the three lifecourse SEP, the disadvantaged category was used as the reference.

Given the large population of E3N, even the smallest deviation can lead to a statistical violation of the proportional risk assumptions as we observed for some variables; nevertheless, no trend seems to emerge looking at residuals graphically. So we used all variables as described previously. (Additional file 4).

#### Lifecourse analyses

To mimic lifecourse experiences, we sequentially adjusted for each SEP resulting in four time-sequenced models as:

- Model A: age + father’s occupation
- Model B: Model A + education
- Model C: Model A + education + occupation
- Model D: Model A + education + occupation + covariates

#### Social mobility

A multiplicative interaction term was introduced for the father’s occupation and the adult occupation, hence defining 5 classes: ‘Stable disadvantaged SEP’ (reference); ‘Downward mobility’; ‘Stable medium SEP’; ‘Upward mobility’; ‘Stable advantaged SEP’.

#### Missing data

To control for possible bias due to missing data, they were imputed using multiple imputation in the overall population with the MICE R package [25]. Fifteen imputations were conducted taking the missing-at-random assumption. All variables tested in the first study step were imputed, including lifecourse SEP. We used Rubin’s combination rules to obtain Cox regression estimates from the multiple imputed data.

#### Sensitivity analyses

We restricted our analyses to women with complete information on SEP and covariates to test the robustness of our results (*N* = 39,122). To take into account potential specificities of invasive BC compared to in situ BC, we also ran Cox proportional hazards regression in women with invasive BC only (87.6% of all incident BC cases in E3N).

### Replication analyses in EPIC-Italy

The same multivariate, lifecourse, and social mobility analyses were replicated in EPIC-Italy. Baseline model (Model1) in EPIC-Italy was further adjusted for center because of the study design. Multiple imputations were also performed in the overall population.

Statistical analyses were performed using R. version 1.2.1114 within R studio version 1.2.5001.

## Results

### E3N cohort

From the entire cohort, women with prevalent cancer, those with another cancer than BC as well as those with missing or inconsistent data on date or status of diagnosis were excluded from the analyses leaving a total of 83,436 women.

Selected characteristics for E3N by BC status are provided in Table 1. During a 24.4-year median follow-up time, 7877 women were diagnosed with BC. Compared to women who did not develop BC, cases were more likely to have an advantaged SEP along the lifecourse, to be older, to be heavy drinkers, to have a high adherence to a Western diet pattern, to be inactive or have low physical activity, and to be in the highest tercile of height and weight. Women diagnosed with BC were also more likely to have a family history of a hormone-related cancer, to be MHT ever user, to have fewer children, to have been older at first childbirth, not to have breastfed, to have a longer reproductive lifespan, to be in pre-menopause at baseline, and to have ever participated in mammographic screening before inclusion. Characteristics by SEP are available in Additional file 5).

**Table 1:** Characteristics of women with available data from E3N according to BC status

Variables	BC		***p***
	**No**	**Yes**	
**Father s occupation (3 cl)**	*n* = 55,766	*n* = 5957	0.025
Advantaged, *n*(%)	9294 (16.67)	1075 (18.05)	
Medium, *n*(%)	23,619 (42.35)	2492 (41.83)	
Disadvantaged, *n*(%)	22,853 (40.98)	2390 (40.12)	
**Education (3 cl)**	*n* = 72,773	*n* = 7577	< 0.001
High, *n*(%)	26,096 (35.86)	2913 (38.45)	
Middle, *n*(%)	36,422 (50.05)	3745 (49.43)	
Low, *n*(%)	10,255 (14.09)	919 (12.13)	
**Occupation (3 cl)**	*n* = 62,726	*n* = 6700	0.017
Advantaged, *n*(%)	10,962 (17.48)	1250 (18.66)	
Medium, *n*(%)	40,679 (64.85)	4332 (64.66)	
Disadvantaged, *n*(%)	11,085 (17.67)	1118 (16.69)	
**Age**	*n* = 75,559	*n* = 7877	< 0.001
< 47.9y, *n*(%)	39,491 (52.27)	3837 (48.71)	
≥ 47.9y, *n*(%)	36,068 (47.73)	4040 (51.29)	
**Alcohol consumption**	*n* = 71,357	*n* = 7455	< 0.001
Abstainer, *n*(%)	7040 (9.87)	678 (9.09)	
Moderate, *n*(%)	38,720 (54.26)	4167 (55.9)	
High, *n*(%)	10,975 (15.38)	1356 (18.19)	
Not responding to Q3, *n*(%)	14,622 (20.49)	1254 (16.82)	
**Smoking status**	*n* = 75,333	*n* = 7850	0.693
Never, *n*(%)	50,531 (67.08)	5269 (67.12)	
Former, *n*(%)	15,204 (20.18)	1560 (19.87)	
Ever, *n*(%)	9598 (12.74)	1021 (13.01)	
**Western diet pattern**	*n* = 71,358	*n* = 7455	< 0.001
[−2.93,-0.511], *n*(%)	18,876 (26.45)	1921 (25.77)	
(−0.511,0.307], *n*(%)	18,868 (26.44)	2089 (28.02)	
(0.307,7.78], *n*(%)	18,991 (26.61)	2191 (29.39)	
Not responding to Q3, *n*(%)	14,623 (20.49)	1254 (16.82)	
**Physical activity (MET)**	*n* = 74,540	*n* = 7768	< 0.001
(7.57,35.9], *n*(%)	25,040 (33.59)	2395 (30.83)	
(4.66,7.57], *n*(%)	24,635 (33.05)	2630 (33.86)	
[0,4.66], *n*(%)	24,865 (33.36)	2743 (35.31)	
**Height**	*n* = 74,316	*n* = 7759	< 0.001
[135,160], *n*(%)	33,983 (45.73)	3355 (43.24)	
(160,164], *n*(%)	16,551 (22.27)	1732 (22.32)	
(164,190], *n*(%)	23,782 (32)	2672 (34.44)	
**Weight**	*n* = 73,847	*n* = 7715	0.076
[29,55], *n*(%)	25,135 (34.04)	2530 (32.79)	
(55,62], *n*(%)	24,737 (33.5)	2611 (33.84)	
(62,163], *n*(%)	23,975 (32.47)	2574 (33.36)	
**Previous ovary cancer (1grade)**	*n* = 75,559	*n* = 7877	0.002
No, *n*(%)	74,709 (98.88)	7757 (98.48)	
Yes, *n*(%)	850 (1.12)	120 (1.52)	
**Previous breast cancer (1grade)**	*n* = 73,693	*n* = 7673	< 0.001
No, *n*(%)	31,622 (42.91)	3039 (39.61)	
Yes, *n*(%)	5240 (7.11)	886 (11.55)	
Not available, *n*(%)	36,831 (49.98)	3748 (48.85)	
**MHT use**	*n* = 67,245	*n* = 7111	< 0.001
No, *n*(%)	58,868 (87.54)	6021 (84.67)	
Yes, *n*(%)	6029 (8.97)	806 (11.33)	
Undefined, *n*(%)	2348 (3.49)	284 (3.99)	
**Number of full term pregnancy**	*n* = 75,551	*n* = 7877	< 0.001
3+, *n*(%)	22,212 (29.4)	2000 (25.39)	
1–2, *n*(%)	44,491 (58.89)	4827 (61.28)	
0, *n*(%)	8848 (11.71)	1050 (13.33)	
**Age at the first childbirth**	*n* = 73,018	*n* = 7660	< 0.001
[14, 23], *n*(%)	26,390 (36.14)	2445 (31.92)	
(23,26], *n*(%)	19,487 (26.69)	1959 (25.57)	
(26,59], *n*(%)	18,293 (25.05)	2206 (28.8)	
No preg, *n*(%)	8848 (12.12)	1050 (13.71)	
**Combined age and number of pregnancy**	*n* = 73,016	*n* = 7660	< 0.001
Early first birth and high number of children, *n*(%)	11,119 (15.23)	938 (12.25)	
High number of children, *n*(%)	10,150 (13.9)	997 (13.02)	
Late first birth and few number of children, *n*(%)	14,575 (19.96)	1794 (23.42)	
Low number of children, *n*(%)	28,324 (38.79)	2881 (37.61)	
No pregnancy, *n*(%)	8848 (12.12)	1050 (13.71)	
**Breastfeeding**	*n* = 67,708	*n* = 7189	0.019
Yes, *n*(%)	40,113 (59.24)	4156 (57.81)	
No, *n*(%)	27,595 (40.76)	3033 (42.19)	
**Reproductive lifespan**	*n* = 69,697	*n* = 7508	< 0.001
[27; 41[, *n*(%)	56,083 (80.47)	5964 (79.44)	
< 27, *n*(%)	1161 (1.67)	80 (1.07)	
≥ 41, *n*(%)	12,453 (17.87)	1464 (19.5)	
**Breast cancer screening**	*n* = 75,559	*n* = 7877	< 0.001
Yes, *n*(%)	52,720 (69.77)	6012 (76.32)	
No, *n*(%)	22,839 (30.23)	1865 (23.68)	
**Postmenopausal status**	*n* = 71,368	*n* = 7675	0.016
Pre-menopausal, *n*(%)	41,683 (58.41)	4593 (59.84)	
Post-menopausal, *n*(%)	29,685 (41.59)	3082 (40.16)	

*P*-values are estimated with log rank tests

### Association between SEP and the risk of BC

Women whose fathers had an advantaged SEP had a higher BC risk compared with women whose fathers had a disadvantaged SEP (M1: HR [95%CI] = 1.10 [1.02, 1.18], Table 2a). Associations were slightly attenuated when adjusting for health behaviours and anthropometric characteristics (HR [95%CI] = 1.08 [1.01, 1.16]), reproductive factors (HR [95%CI] = 1.05 [0.98, 1.13]), or for all covariates (M2, HR [95%CI] = 1.04 [0.97, 1.12]). Women whose fathers had a medium SEP were not at higher risk for BC.

**Table 2:** Cox proportional hazard regression of BC risk using data from multiple imputation in E3N (*N* = 83,436)

		A. Father’s occupation^**a**^		B. Education^**b**^		C. Occupation^**a**^	
		**Medium**	**Advantaged**	**Middle**	**High**	**Medium**	**Advantaged**
		HR [95%CI]	HR [95%CI]	HR [95%CI]	HR [95%CI]	HR [95%CI]	HR [95%CI]
**M1**		1.00 [0.95, 1.06]	1.10 [1.02, 1.18]	1.09 [1.01, 1.17]	1.21 [1.12, 1.30]	1.02 [0.96, 1.09]	1.08 [1.00, 1.17]
Health behaviours And Anthropometric factors	M1 + Alcohol consumption	1.00 [0.95, 1.06]	1.09 [1.02, 1.17]	1.09 [1.02, 1.17]	1.20 [1.11, 1.29]	1.02 [0.96, 1.09]	1.08 [1.00, 1.17]
	M1 + Smoking status	1.00 [0.95, 1.06]	1.10 [1.02, 1.18]	1.09 [1.02, 1.17]	1.21 [1.12, 1.30]	1.03 [0.96, 1.09]	1.08 [1.00, 1.17]
	M1 + Western diet pattern	1.00 [0.95, 1.06]	1.10 [1.02, 1.18]	1.10 [1.02, 1.18]	1.21 [1.13, 1.31]	1.03 [0.97, 1.09]	1.09 [1.00, 1.18]
	M1 + Physical activity	1.00 [0.94, 1.05]	1.09 [1.02, 1.17]	1.08 [1.00, 1.16]	1.18 [1.10, 1.28]	1.02 [0.96, 1.08]	1.07 [0.99, 1.16]
	M1 + Height	1.00 [0.94, 1.05]	1.09 [1.01, 1.17]	1.09 [1.01, 1.17]	1.19 [1.11, 1.28]	1.02 [0.96, 1.08]	1.07 [0.99, 1.16]
	M1 + Weight	1.00 [0.95, 1.06]	1.10 [1.02, 1.18]	1.10 [1.02, 1.18]	1.21 [1.13, 1.31]	1.03 [0.97, 1.09]	1.08 [1.00, 1.17]
**M1 + all health behaviours and anthropometrics factors**		1.00 [0.94, 1.05]	1.08 [1.01, 1.16]	1.08 [1.00, 1.16]	1.18 [1.09, 1.27]	1.02 [0.96, 1.08]	1.07 [0.99, 1.16]
Reproductive factors	M1 + Family history of ovarian cancer	1.00 [0.95, 1.06]	1.10 [1.02, 1.18]	1.09 [1.01, 1.17]	1.20 [1.12, 1.30]	1.02 [0.96, 1.09]	1.08 [1.00, 1.17]
	M1 + Family history of breast cancer	1.00 [0.94, 1.05]	1.09 [1.01, 1.17]	1.09 [1.01, 1.17]	1.19 [1.11, 1.29]	1.02 [0.96, 1.09]	1.08 [1.00, 1.17]
	M1 + MHT use	1.00 [0.95, 1.06]	1.10 [1.02, 1.18]	1.08 [1.01, 1.16]	1.20 [1.11, 1.29]	1.02 [0.96, 1.08]	1.08 [0.99, 1.16]
	M1 + Age at the first childbirth	0.99 [0.94, 1.05]	1.07 [1.00, 1.15]	1.07 [1.00, 1.15]	1.13 [1.04, 1.22]	1.01 [0.95, 1.07]	1.07 [0.98, 1.16]
	M1 + Breastfeeding	1.00 [0.95, 1.06]	1.10 [1.02, 1.18]	1.09 [1.02, 1.17]	1.21 [1.12, 1.30]	1.03 [0.97, 1.09]	1.08 [1.00, 1.17]
	M1 + Reproductive lifespan	1.00 [0.94, 1.05]	1.09 [1.02, 1.17]	1.08 [1.01, 1.16]	1.19 [1.11, 1.29]	1.02 [0.96, 1.08]	1.07 [0.99, 1.16]
	M1 + BC screening	1.00 [0.94, 1.05]	1.09 [1.02, 1.17]	1.08 [1.00, 1.16]	1.19 [1.10, 1.28]	1.02 [0.96, 1.08]	1.08 [1.00, 1.17]
	M1 + Postmenopausal status	1.00 [0.95, 1.06]	1.10 [1.02, 1.18]	1.08 [1.01, 1.16]	1.19 [1.11, 1.28]	1.02 [0.96, 1.08]	1.08 [1.00, 1.17]
**Model 1 + all reproductive factors**		0.98 [0.93, 1.04]	1.05 [0.98, 1.13]	1.03 [0.96, 1.11]	1.06 [0.99, 1.15]	0.99 [0.93, 1.05]	1.06 [0.98, 1.15]
**M2**		0.98 [0.93, 1.04]	1.04 [0.97, 1.12]	1.03 [0.96, 1.11]	1.06 [0.98, 1.14]	0.99 [0.93, 1.05]	1.05 [0.97, 1.14]

M1 is adjusted for age

^a^Referent group: “Disadvantaged”

^b^Referent group: “Low education”

M2 is fully adjusted model

Hazard ratio (HR) and confidence interval are reported for (A) father’s occupation (B) education and (C) occupation

We found a positive association between women’s education and BC risk (M1: HR [95%CI] = 1.21 [1.12, 1.30], Table 2b). Controlling for health behaviours and anthropometric characteristics only slightly attenuated the associations (HR [95%CI] = 1.18 [1.09, 1.27]) while controlling for reproductive factors, in particular age at first childbirth, affected it more strongly (HR[95%CI] = 1.06 [0.99, 1.15]). HRs were further reduced after controlling for all covariates (M2: HR [95%CI] = 1.06 [0.98, 1.14]).

Compared with their disadvantaged counterparts, women with an advantaged occupation had a higher risk of BC (HR[95%CI] = 1.08 [1.00, 1.17], Table 2c). HRs were slightly attenuated after adjustment for either each block of covariates or both (M2, HR[95%CI] = 1.05 [0.97, 1.14], Table 2c). No evidence of an increased risk was observed for women with medium SEP.

Regarding the impact of other covariates on the risk of BC, higher age, high alcohol consumption, higher adherence to a Western diet pattern, lower physical activity, higher height, a family history of hormone-related cancer, use of MHT, and higher age at first childbirth were associated with a higher risk of BC in the fully adjusted model. Inversely, women with a shorter reproductive lifespan, no adherence to BC screening, and who were in post-menopause had a lower risk of BC (Additional file 6).

### Lifecourse SEP and the risk of BC

When we sequentially adjusted for each lifecourse SEP, only education was associated with BC risk. HRs for education were not affected when adjusting for childhood SEP (Model B, HR[95%CI] = 1.19 [1.11; 1.29], Table 3) or both childhood and adulthood SEP (Model C, HR[95%CI] = 1.23 [1.12; 1.35], Table 3). The association was attenuated after adjustment for age at first childbirth (HR[95%CI] = 1.14 [1.04; 1.25], data not shown) and HRs were weakened in the fully adjusted model (Model D, R[95%CI] = 1.06 [0.97; 1.17], Table 3).

**Table 3:** Lifecourse multiple regression analyses of SEP with the risk of BC in E3N using imputed data (*N* = 83,436)

			E3N			
			Model A	Model B	Model C	Model D
**Covariates**	**Reference**	**Modality**	HR [95%CI]	HR [95%CI]	HR [95%CI]	HR [95%CI]
Father’s occupation	Disadvantaged	Medium	1.00 [0.95; 1.06]	0.98 [0.93; 1.04]	0.98 [0.93; 1.04]	0.98 [0.93; 1.04]
		Advantaged	1.10 [1.02; 1.18]	1.06 [0.98; 1.14]	1.05 [0.98; 1.13]	1.03 [0.96; 1.11]
Education	Low	Middle	–	1.09 [1.01; 1.17]	1.12 [1.02; 1.22]	1.04 [0.95; 1.14]
		High	–	1.19 [1.11; 1.29]	1.23 [1.12; 1.35]	1.06 [0.97; 1.17]
Occupation	Disadvantaged	Medium	–	–	0.95 [0.88; 1.02]	0.97 [0.90; 1.04]
		Advantaged	–	–	1.00 [0.91; 1.09]	1.03 [0.94; 1.13]

Model A is adjusted for age and father’s occupation

Model B is adjusted for age, father’s occupation and education

Model C is adjusted for age and both SEP

Model D is adjusted for age, both SEP and all covariates (i.e. alcohol consumption, smoking status, physical activity, Western diet pattern, height, weight, family history of ovarian cancer or BC, MHT use, breastfeeding, cancer screening, reproductive lifespan, age at first childbirth and menopausal status)

### Effect of social mobility on the risk of BC

Women who experienced a stable advantaged SEP had a higher risk of BC than those with a stable disadvantaged SEP (Model1: HR[95%CI] = 1.24 [1.07; 1.43], Table 4). HR estimates were attenuated, especially after adjustment for age at first childbirth (HR[95%CI] = 1.19 [1.03; 1.37, data not shown), and after adjustment for all covariates (Fully adjusted model: HR[95%CI] = 1.13 [0.98; 1.31], Table 4).

**Table 4:** Association of social mobility with the risk of BC in E3N using imputed data (*N* = 83,436)

			E3N			
			Model 1	Model 1 + HB-A	Model 1 + RF	Fully adjusted model
**Covariates**	**Reference**	**Modality**	HR [95%CI]	HR [95%CI]	HR [95%CI]	HR [95%CI]
Social mobility	Stable disadvantaged SEP	Downward mobility	1.06 [0.95; 1.19]	1.04 [0.93; 1.17]	1.00 [0.90; 1.12]	1.00 [0.89; 1.12]
		Stable medium SEP	1.02 [0.92; 1.12]	1.01 [0.91; 1.11]	0.96 [0.87; 1.06]	0.96 [0.87; 1.06]
		Upward mobility	1.04 [0.94; 1.14]	1.03 [0.93; 1.13]	1.00 [0.91; 1.10]	0.99 [0.90; 1.09]
		Stable advantaged SEP	1.24 [1.07; 1.43]	1.20 [1.04; 1.39]	1.15 [0.99; 1.33]	1.13 [0.98; 1.31]

Model 1 is adjusted for age and social mobility

Model 1 + HB-A is adjusted for age, social mobility, alcohol consumption, smoking status, physical activity, Western diet pattern, height, weight

Model 1 + RF is adjusted for age, social mobility, family history of ovarian cancer or BC, MHT use, breastfeeding, cancer screening, reproductive lifespan, age at first childbirth and menopausal status

Fully adjusted model is adjusted for age, social mobility and all covariates

### Sensitivity analyses

Complete cases analyses showed similar results although associations with BC risk were slightly stronger for education and occupation (Additional file 7). HR estimates for education were weakened but not entirely explained after accounting for all covariates. A similar pattern was observed with participants’ occupation. When we restricted our analyses to women who developed an invasive BC, results were comparable (Additional file 8).

### External validation in EPIC-Italy

Results in EPIC-Italy are provided in Additional file 9, 10, 11, 12. Briefly, HRs for the risk of BC in highly educated women were similar to those observed in E3N (Model 1: HR[95%CI] = 1.19 [0.96; 1.47], Additional file 10). Adjustment for age at first childbirth attenuated mostly the relation (HR[95%CI] = 1.05 [0.84; 1.31]). Association between education and the risk of BC was not affected by other SEP indicators as we observed for E3N (Model C: HR[95%CI] = 1.18 [0.94; 1.49], Additional file 11). Women with a stable medium or stable advantaged SEP had a higher risk of BC compared to stable disadvantaged SEP, and HRs were marginally affected after adjustment for covariates (Additional file 12).

## Discussion

In a large prospective cohort with available lifecourse SEP and a wide array of covariates, we found that women with an advantaged SEP had a higher risk of BC at each considered time point. Lifecourse analyses suggested that education had a stronger effect on BC risk. Analyses on social mobility indicated that women who stay in a stable advantaged SEP had a higher risk of BC than those remaining in stable disadvantaged SEP. Associations were weaker when adjusting for reproductive factors. Age at first childbirth was the strongest contributor to SEP-associated BC risk. Adjustment for BC screening participation or for health behaviours and anthropometric characteristics only marginally modified the association between education and BC risk. Results were robust to the sensitivity analyses we performed and strengthened by the external validation in EPIC-Italy.

Our study adds to the literature on the effect of lifecourse SEP on the risk of BC. In agreement with previous studies, education was the SEP indicator most strongly related to BC risk [9, 14, 17] and the association was mainly weakened by reproductive factors [9–15]. But we also show that this impact persists even after considering the main other potential mediators, which constitutes an original finding of our work.

Studies on the etiology of BC have highlighted the major role of BC risk factors such as weight at birth, early age to first menstruation, alcohol consumption, age at first childbirth [26], or late age at menopause [27]. All those factors are related, to various extents, to hormonal pathways. Our study supports the importance of age at first childbirth, corresponding to the time when maturation of the breast tissue ends [28], on BC risk.

We initially hypothesized that the remaining association after adjustment for reproductive factors could be explained by the fact that higher educated women are more likely to participate in mammographic screening [29]. But we observed only a modest effect on risk by adjusting for BC screening, in agreement with previous studies [13, 18]. Adjustment for health behaviours and anthropometric characteristics also had a modest effect on the association between education and BC risk.

According to the literature, some of the hormone-related risk factors for BC occur early in life, such as birth weight or age at menarche, and have been found to be associated with SEP [30–32]. This suggests that SEP in early life could be important. Our results are not in favour of the hypothesis of a socially differentiated early sensitive period in BC risk. Results on social mobility show that women in an advantaged SEP throughout their lives are the most at risk group, suggesting that these women would cumulate harmful effects over the lifecourse. Additional and more specific analyses are needed to better define the impact of risk factors accumulation across the lifecourse.

This work was conducted on a large prospective cohort of French women in which a very large number and breadth of potential mediators were available. The prospective design limits both recall and reverse causation biases. Women have been followed since the 1990s until now, which allows us to have a long view on the disease development. Self-reported cancer cases were validated avoiding misclassification. Additionally, the use of a second prospective and independent cohort, EPIC-Italy, enabled us to replicate our findings, providing an external validation.

The main limitation in E3N lies in its recruitment especially when focusing on social inequalities. Women volunteers included in this study were all affiliated with a national health system (MGEN) that mainly insures people working in the French education system and spouses. Although the cohort also includes administrative and cleaning staff, the average educational level of the cohort is higher compared to the one in the general population. However, by considering SEP at different life periods from childhood, we were able to observe a certain degree of variability. There is likely to be heterogeneity, measurement and misclassification errors in both cohorts regarding the 3 life course SEP indicators. However these individual-based measurements of SEP could capture individual factors (e.g. material, behavioural, or psychosocial factors), provide information about individuals’ accesses to social and economic resources, and be related to macro-environmental features (e.g. geographical location). We cannot rule out selection bias due to attrition and loss of follow-up. To allow for uncertainty about the missing data, we ran multiple imputations and analyses on complete cases, which provided consistent results. Even if we considered one of the largest set of covariates, it is still possible that other factors may contribute in the relationship between SEP and BC. In particular, breast density appears to be an interesting risk factors of BC to consider [33, 34]. Several studies have reported an independent association between SEP, including education, and breast density after accounting for the potential mediators we considered in our study. Breast density is suspected to modulate estrogen level which could be at the origin of the risk of BC. We could think that breast density may reflect another path modifying level of estrogens that has not been taken into account here. Alternatively, other mechanisms could be investigated, including the impact of perceived stress on levels of biomarkers suspected to be involved in BC risk [35–38]. The approach we used in our study allowed us to identify potential mediators from a large range of factors. A better understanding of the causal pathways through which educational processes operate is now needed using causal approaches.

## Conclusion

In the E3N cohort, women with advantaged SEP along the life course had an increased risk of BC. Among the three investigated SEP time points, education was the factor most strongly related to subsequent BC risk. Accounting for the large set of mediators we studied, age at first childbirth explained an important part of the observed association between SEP and BC risk. Other potential mediators, including BC screening, had a lesser effect on the association. The association between life course SEP and BC risk was not fully explained suggesting that further research is needed to identify additional mediators. The association between a stable advantaged SEP and BC risk suggests a cumulative damaging effect of advantaged SEP across the life course. Our results from an independent cohort from EPIC-Italy were consistent in terms of direction and size of the effect compared to those obtained in E3N, although with less power due to reduced cohort size. Finally, studies need to be develop to identify the causal mechanisms of a higher BC risk in women with advantaged SEP over the lifecourse.

## Supplementary Information

**Additional file 1.** Cohorts’ description.

**Additional file 2.** Coding specificities.

**Additional file 3.** Description of all covariates selected according to the literature and tested in bivariate analyses.

**Additional file 4.** Scatterplots of Schoenfeld residues for variables that do not respect the proportional risk assumption.

**Additional file 5.** Characteristics of women with available data from E3N according to SEP.

**Additional file 6 **Forestplot of the association of the three time point SEP and each covariate used in the fully adjusted model in E3N (*N* = 83,436).

**Additional file 7 **Association between life course SEP on the risk of BC using complete cases in E3N [*N* = 39,122].

**Additional file 8 **Association between life course SEP on the risk of invasive BC only using imputed data in E3N (*N* = 82,458).

**Additional file 9.** Characteristics of women with available data from EPIC-Italy according to BC status and by SEP.

**Additional file 10 **Cox proportional hazard regression of BC risk using data from multiple imputation in EPIC-Italy (*N* = 20,530).

**Additional file 11.** Lifecourse multiple regression analyses of SEP with the future risk of BC in EPIC-Italy using imputed data [N = 20,530].

**Additional file 12.** Association of social mobility with the risk of BC in EPIC-Italy using imputed data [N = 20,530].

## Data Availability

The datasets generated and/or analysed during the current study are not publicly available and permission to use the data is restricted to the teams in charge of the cohorts, which can be extended to collaborators with a specific research agreement.

## Abbreviations

BC: Breast cancer
HR: Hazard ratio
MHT: Menopausal hormone therapy
SEP: Socioeconomic position

## References

[1] Hvidtfeldt UA. Mechanisms underlying social inequality in post-menopausal breast cancer. Dan Med J. 2014;61(10):B4922. 25283627

[2] Lundqvist A, Andersson E, Ahlberg I, Nilbert M, Gerdtham U (2016). Socioeconomic inequalities in breast cancer incidence and mortality in Europe-a systematic review and meta-analysis. Eur J Pub Health.

[3] dos Santos Silva I, Beral V. Socioeconomic differences in reproductive behaviour. IARC Sci Publ. 1997;(138):285–308. 9353670

[4] Cavelaars AE, Kunst AE, Geurts JJ, Crialesi R, Grötvedt L, Helmert U (2000). Persistent variations in average height between countries and between socio-economic groups: an overview of 10 European countries. Ann Hum Biol.

[5] Barriuso L, Miqueleiz E, Albaladejo R, Villanueva R, Santos JM, Regidor E (2015). Socioeconomic position and childhood-adolescent weight status in rich countries: a systematic review, 1990-2013. BMC Pediatr.

[6] Petrovic D, de Mestral C, Bochud M, Bartley M, Kivimäki M, Vineis P (2018). The contribution of health behaviors to socioeconomic inequalities in health: a systematic review. Prev Med.

[7] Martikainen P, Brunner E, Marmot M (2003). Socioeconomic differences in dietary patterns among middle-aged men and women. Soc Sci Med 1982.

[8] Hussain SK, Altieri A, Sundquist J, Hemminki K (2008). Influence of education level on breast cancer risk and survival in Sweden between 1990 and 2004. Int J Cancer.

[9] Pudrovska T, Anikputa B (2012). The role of early-life socioeconomic status in breast Cancer incidence and mortality: unraveling life course mechanisms. J Aging Health.

[10] Larsen SB, Olsen A, Lynch J, Christensen J, Overvad K, Tjønneland A (2011). Socioeconomic position and lifestyle in relation to breast cancer incidence among postmenopausal women: a prospective cohort study, Denmark, 1993–2006. Cancer Epidemiol.

[11] Villeneuve S, Févotte J, Anger A, Truong T, Lamkarkach F, Gaye O (2011). Breast cancer risk by occupation and industry: analysis of the CECILE study, a population-based case-control study in France. Am J Ind Med.

[12] Menvielle G, Kunst AE, van Gils CH, Peeters PH, Boshuizen H, Overvad K (2011). The contribution of risk factors to the higher incidence of invasive and in situ breast cancers in women with higher levels of education in the European prospective investigation into cancer and nutrition. Am J Epidemiol.

[13] Braaten T, Weiderpass E, Kumle M, Lund E (2005). Explaining the socioeconomic variation in cancer risk in the Norwegian women and Cancer study. Cancer Epidemiol Biomark Prev Publ Am Assoc Cancer Res Cosponsored Am Soc Prev Oncol.

[14] Danø H, Hansen KD, Jensen P, Petersen JH, Jacobsen R, Ewertz M (2004). Fertility pattern does not explain social gradient in breast cancer in Denmark. Int J Cancer.

[15] Heck KE, Pamuk ER (1997). Explaining the relation between education and postmenopausal breast cancer. Am J Epidemiol.

[16] Beiki O, Hall P, Ekbom A, Moradi T (2012). Breast cancer incidence and case fatality among 4.7 million women in relation to social and ethnic background: a population-based cohort study. Breast Cancer Res BCR.

[17] Carlsen K, Høybye MT, Dalton SO, Tjønneland A (2008). Social inequality and incidence of and survival from breast cancer in a population-based study in Denmark, 1994–2003. Eur J Cancer Oxf Engl 1990.

[18] Meijer M, Bloomfield K, Engholm G (2013). Neighbourhoods matter too: the association between neighbourhood socioeconomic position, population density and breast, prostate and lung cancer incidence in Denmark between 2004 and 2008. J Epidemiol Community Health.

[19] van der Linden BWA, Courvoisier DS, Cheval B, Sieber S, Bracke P, Guessous I (2018). Effect of childhood socioeconomic conditions on cancer onset in later life: an ambidirectional cohort study. Int J Public Health..

[20] Clavel-Chapelon F (2015). Cohort profile: the French E3N cohort study. Int J Epidemiol.

[21] Palli D, Berrino F, Vineis P, Tumino R, Panico S, Masala G, et al. A molecular epidemiology project on diet and cancer: the EPIC-Italy Prospective Study. Design and baseline characteristics of participants. Tumori. 2003;89(6):586–93. 10.1177/030089160308900602 14870823

[22] d’Errico A, Ricceri F, Stringhini S, Carmeli C, Kivimaki M, Bartley M (2017). Socioeconomic indicators in epidemiologic research: a practical example from the LIFEPATH study. PLoS One.

[23] Wareham NJ, Jakes RW, Rennie KL, Schuit J, Mitchell J, Hennings S (2003). Validity and repeatability of a simple index derived from the short physical activity questionnaire used in the European prospective investigation into Cancer and nutrition (EPIC) study. Public Health Nutr.

[24] Agnoli C, Krogh V, Grioni S, Sieri S, Palli D, Masala G (2011). A priori-defined dietary patterns are associated with reduced risk of stroke in a large Italian cohort. J Nutr.

[25] Buuren S van, Groothuis-Oudshoorn CGM. mice: Multivariate Imputation by Chained Equations in R. J Stat Softw [Internet]. 2011 [cited 2019 Dec 18];45(3). Available from: https://research.utwente.nl/en/publications/mice-multivariate-imputation-by-chained-equations-in-r.

[26] Colditz GA, Bohlke K, Berkey CS (2014). Breast cancer risk accumulation starts early – prevention must also. Breast Cancer Res Treat.

[27] Colditz GA, Rosner B (2000). Cumulative risk of breast cancer to age 70 years according to risk factor status: data from the nurses’ health study. Am J Epidemiol.

[28] Russo J, Tay LK, Russo IH (1982). Differentiation of the mammary gland and susceptibility to carcinogenesis. Breast Cancer Res Treat.

[29] Willems B, Bracke P (2018). The education gradient in cancer screening participation: a consistent phenomenon across Europe?. Int J Public Health.

[30] Rafique M, Zia S, Ubaidullah, Sultan MA. Impact of socioeconomic status on birth weight and length of Newborns delivered at Services Hospital Lahore. Pak Paed J. 2008;32(2):94-100.

[31] Khalid H, Khawar K, Fawad M, Farhat M, Imran M, Shahnawaz M, et al. Age of Menarche in Relation to Socioeconomic Status, BMI, Physical Activity and Stress Among High School Girls.: 7.

[32] Schoenaker DA, Jackson CA, Rowlands JV, Mishra GD (2014). Socioeconomic position, lifestyle factors and age at natural menopause: a systematic review and meta-analyses of studies across six continents. Int J Epidemiol.

[33] Samuels L, Harkness E, Astley SM, Maxwell A, Sergeant J, Morris J, et al. The Relationship of Volumetric Breast Density to Socio-Economic Status in a Screening Population. In: Fujita H, Hara T, Muramatsu C, editors. Breast Imaging. Cham: Springer International Publishing; 2014. p. 273–281. (Lecture Notes in Computer Science).

[34] Aitken Z, Walker K, Stegeman BH, Wark PA, Moss SM, McCormack VA (2010). Mammographic density and markers of socioeconomic status: a cross-sectional study. BMC Cancer.

[35] Lennartsson A-K, Theorell T, Rockwood AL, Kushnir MM, Jonsdottir IH. Perceived Stress at Work Is Associated with Lower Levels of DHEA-S. PLoS ONE [Internet]. 2013 Aug 28 [cited 2020 Jan 14];8(8). Available from: https://www.ncbi.nlm.nih.gov/pmc/articles/PMC3756071/. 10.1371/journal.pone.0072460 PMC3756071 24015247

[36] Cho S, Park W-J, Kang W, Lim H-M, Ahn J-S, Lim D-Y, et al. The association between serum dehydroepiandrosterone sulfate (DHEAS) levels and job-related stress among female nurses. Ann Occup Environ Med [Internet]. 2019 15 [cited 2020 Jan 14];31(1). Available from: 10.35371/aoem.2019.31.e18. 10.35371/aoem.2019.31.e18 PMC6779852 31620295

[37] Kaaks R, Rinaldi S, Key TJ, Berrino F, Peeters PHM, Biessy C (2005). Postmenopausal serum androgens, oestrogens and breast cancer risk: the European prospective investigation into cancer and nutrition. Endocr Relat Cancer.

[38] Pudrovska T (2013). Job authority and breast Cancer. Soc Forces Sci Medium Soc Study Interpret.

