# Supplementary material for: The impact of lifecourse socio-economic position and individual social mobility on breast cancer risk
Source: BMC Cancer. 2020 Nov 23;20:1138. doi: 10.1186/s12885-020-07648-w (PMC7684912; doi:10.1186/s12885-020-07648-w)
Supplement: Supplementary file 1 — Additional file 1. Cohorts’ description. [file 12885_2020_7648_MOESM1_ESM.docx]

Cohorts’ description

| **Cohort** | **Description** | **Selection** | **Ethics statement** |
| --- | --- | --- | --- |
| **E3N**  **(N = 98,995)** | MGEN is a national health insurance plan that primarily covers people working for the national education system. At inclusion, participants have filled the first questionnaire to collect information about anthropometric measures, lifestyle / behaviours, SEP and health, and gave written informed consent for the study. Self-administered questionnaires are sent every 2 years since 1990. In the third questionnaire (Q3 - June 1993), special questionnaire about diet was dispensed. There was a double identification of cancer cases: first self-reported by women and then confirmed with physicians or medical reports. | Among the original E3N cohort, 6,909 women with a first cancer before inclusion were excluded, as well as 8,650 women with a diagnosis of cancer other than breast cancer. The resulting study sample included 83,436 women. | The study was approved by the French Commission for Data Protection and Privacy (NCT03285230, CNIL no. 327346 V 13). |
| **EPIC-Italy**  **(N = 34,152)** | Participants were from Turin and Varese classified as Northern Italy and Naples and Ragusa as Southern Italy. At inclusion, participants have filled a questionnaire to collect information about anthropometric measures, lifestyle / behaviours, SEP and health. Cancer cases were identified through automated linkages to cancer and mortality registries or confirmed with histological reports, hospital discharge systems or collected through periodic personal contact. | Among the original EPIC-Italy cohort, 4 participants were excluded due to missing data on time of cancer follow up; 11,654 male participants were also excluded, as well as 1634 women with a diagnosis of cancer other than breast cancer, 328 prior to inclusion and 2 with no information on cancer site. The resulting study sample included 20,530 women. | All participants signed a written informed consent and the ethical review boards of the International Agency for Research on Cancer, and of the collaborating institutions responsible for subject recruitment in each of the EPIC recruitment centres approved the study. |
