# Supplementary material for: The impact of lifecourse socio-economic position and individual social mobility on breast cancer risk
Source: BMC Cancer. 2020 Nov 23;20:1138. doi: 10.1186/s12885-020-07648-w (PMC7684912; doi:10.1186/s12885-020-07648-w)
Supplement: Supplementary file 2 — Additional file 2. Coding specificities. [file 12885_2020_7648_MOESM2_ESM.docx]

Coding specificities

**A. Mesure of each SEP**

For father's occupation and occupation, we applied 3 E-SEC categories: less advantaged occupations [lower clerical, services, and sales workers; skilled workers; semi and unskilled workers (Class 7-9 ESEC)]; medium occupation [small employers and self-employed; farmers; lower supervisors and technicians (Class 4, 5, and 6 ESEC)] and more advantaged occupation [higher professionals and managers, lower professionals and managers; higher clerical, services and sales workers (Class 1-3 ESEC)].

For education, we defined 3 categories: low level of education [primary or lower secondary school], medium level [higher secondary school] and high level attainment [tertiary education].

**B. Detailed inversions of SEP classification compared to original classification from d'Errico & al.**

For childhood and adulthood SEP, participants who referred ‘agrégé’ and were classified as ‘small employers and self-employed; farmers; lower supervisors and technicians’ in original classification was reclassified here as ‘higher professionals and managers, lower professionals and managers; higher clerical, services and sales workers’.

For young adulthood SEP, ‘BTEC First Diploma - Youth Training ‘ originally classified as ‘higher secondary school’ was considered here as ‘primary or lower secondary school of education’.

**C. Detailed categories related to diet questionnaire at Q3**

In E3N, information on diet and alcohol consumption were collected at Q3 (near 3 years after inclusion). To avoid losing a large number of women on complete case analyses, those lost to follow up at Q3 who had all the information from Q1 were kept and classified as 'not responding to Q3' for alcohol and dietary patterns.
