# Supplementary material for: The impact of lifecourse socio-economic position and individual social mobility on breast cancer risk
Source: BMC Cancer. 2020 Nov 23;20:1138. doi: 10.1186/s12885-020-07648-w (PMC7684912; doi:10.1186/s12885-020-07648-w)
Supplement: Supplementary file 3 — Additional file 3. Description of all covariates selected according to the literature and tested in bivariate analyses. [file 12885_2020_7648_MOESM3_ESM.docx]

Description of all covariates selected according to the literature and tested in bivariate analyses.

|  |  | **Covariates** | **Modalities** | **Kept for multivariate analyses** |
| --- | --- | --- | --- | --- |
| i) Health behaviours and anthropometric characteristics |  | Alcohol consumption | High (>20 grams per day) | **Yes** |
|  |  |  | Moderate (: ≤20 grams per day but not abstainers) |  |
|  |  |  | Abstainers |  |
|  |  |  | Not responding to Q3* |  |
|  |  | Smoking status | Current | **Yes** |
|  |  |  | Former |  |
|  |  |  | Never |  |
|  |  | Physical activity (MET) | High ((7.57,35.9]) | **Yes** |
|  |  |  | Medium ((4.66;7.57]) |  |
|  |  |  | Low or inactive ((0;4.66]) |  |
|  |  | Western diet pattern | Low adherence ([-2.93,-0.511]) | **Yes** |
|  |  |  | Medium adherence ((-0.511,0.307]) |  |
|  |  |  | High adherence ((0.307,7.78]) |  |
|  |  |  | Not responding to Q3* |  |
|  |  | BMI | Underweight | No** |
|  |  |  | Normal |  |
|  |  |  | Overweight |  |
|  |  |  | Obesity |  |
|  |  | Height (cm) | [135,160] | **Yes** |
|  |  |  | (160,164] |  |
|  |  |  | (164,190] |  |
|  |  | Weight (kg) | [29,55] | **Yes** |
|  |  |  | (55,62] |  |
|  |  |  | (62,163] |  |
| ii) Reproductive factors |  | Age at menarche | <11 | No |
|  |  |  | ≥11 |  |
|  |  | Oral contraceptive | No | No |
|  |  |  | Yes |  |
|  |  | Menopausal hormone therapy | No | **Yes** |
|  |  |  | Yes |  |
|  |  |  | Undefined |  |
|  |  | Number of full term pregnancies | 0 | No |
|  |  |  | 1 - 2 |  |
|  |  |  | 3+ |  |
|  |  | Age at the first childbirth | [14,23] | **Yes** |
|  |  |  | (23,26] |  |
|  |  |  | (26,59] |  |
|  |  |  | No pregnancy |  |
|  |  | Breastfeeding | Yes | **Yes** |
|  |  |  | No |  |
|  |  | Breastfeeding duration (months) | (5.5,120] | No |
|  |  |  | (2.5,5.5] |  |
|  |  |  | [1,2.5] |  |
|  |  |  | No preg - No breastfeed |  |
|  |  | Age at first breastfeeding | [14,23] | No |
|  |  |  | (23,26] |  |
|  |  |  | (26,47] |  |
|  |  |  | No preg - No breastfeed |  |
|  |  | Menopausal status at diagnosis | Pre-menopausal | No |
|  |  |  | Post-menopausal |  |
|  |  | Menopausal status at baseline | Pre-menopausal | **Yes** |
|  |  |  | Post-menopausal |  |
|  |  | Reproductive lifespan*** | <27 | **Yes** |
|  |  |  | [27; 41[ |  |
|  |  |  | ≥41 |  |
|  |  | Number of cycle before first pregnancy | [6.52,130] | No |
|  |  |  | (130,170] |  |
|  |  |  | (170,639] |  |
|  |  |  | No pregnancy |  |
|  | Family history of hormone-related cancer and screening | Family history of ovarian cancer | No | **Yes** |
|  |  |  | Yes |  |
|  |  | Family history of breast cancer | No | **Yes** |
|  |  |  | Yes |  |
|  |  |  | Undefined |  |
|  |  | Breast cancer screening | Yes | **Yes** |
|  |  |  | No |  |
| *Supplementary text 3B | |  |  |  |
| ** Height and weight were used instead of BMI in order to capture the specific effect of each variable. | | | |  |

*** Define from the age of menarche to age of menopause.
