# Supplementary material for: The impact of lifecourse socio-economic position and individual social mobility on breast cancer risk
Source: BMC Cancer. 2020 Nov 23;20:1138. doi: 10.1186/s12885-020-07648-w (PMC7684912; doi:10.1186/s12885-020-07648-w)
Supplement: Supplementary file 5 — Additional file 5. Characteristics of women with available data from E3N according to SEP. [file 12885_2020_7648_MOESM5_ESM.docx]

Characteristics of women with available data from E3N according to SEP.

| **Variables** | **Father’s occupation** | | | | | **Education** | | | | | **Occupation** | | | | |
| --- | --- | --- | --- | --- | --- | --- | --- | --- | --- | --- | --- | --- | --- | --- | --- |
|  | **Missing** | **High** | **Medium** | **Low** | **p** | **Missing** | **High** | **Middle** | **Low** | **p** | **Missing** | **High** | **Medium** | **Low** | **p** |
| **Age*** | 0 | n= 10369 | n= 26111 | n= 25243 | <0.001 | 0 | n= 29009 | n= 40167 | n= 11174 | <0.001 | 0 | n= 12212 | n= 45011 | n= 12203 | <0.001 |
| < 47.9 y, n(%) |  | 5327 (51.37) | 13451 (51.51) | 13798 (54.66) |  |  | 17728 (61.11) | 19712 (49.08) | 4444 (39.77) |  |  | 5107 (41.82) | 25129 (55.83) | 6467 (53) |  |
| ≥ 47.9 y, n(%) |  | 5042 (48.63) | 12660 (48.49) | 11445 (45.34) |  |  | 11281 (38.89) | 20455 (50.92) | 6730 (60.23) |  |  | 7105 (58.18) | 19882 (44.17) | 5736 (47) |  |
| **Alcohol consumption** | 3294 | n= 9783 | n= 24807 | n= 23839 | <0.001 | 4419 | n= 27578 | n= 38193 | n= 10160 | <0.001 | 3966 | n= 11562 | n= 42651 | n= 11247 | <0.001 |
| Abstainer, n(%) |  | 998 (10.2) | 2673 (10.78) | 2524 (10.59) |  |  | 2426 (8.8) | 3980 (10.42) | 1040 (10.24) |  |  | 1205 (10.42) | 4537 (10.64) | 1169 (10.39) |  |
| Moderate, n(%) |  | 5699 (58.25) | 15003 (60.48) | 14366 (60.26) |  |  | 15150 (54.94) | 21431 (56.11) | 4838 (47.62) |  |  | 6891 (59.6) | 25597 (60.02) | 6349 (56.45) |  |
| High, n(%) |  | 1939 (19.82) | 4142 (16.7) | 3917 (16.43) |  |  | 5088 (18.45) | 5640 (14.77) | 1193 (11.74) |  |  | 2068 (17.89) | 7346 (17.22) | 1736 (15.44) |  |
| Not responding to Q3, n(%) |  | 1147 (11.72) | 2989 (12.05) | 3032 (12.72) |  |  | 4914 (17.82) | 7142 (18.7) | 3089 (30.4) |  |  | 1398 (12.09) | 5171 (12.12) | 1993 (17.72) |  |
| **Smoking status** | 103 | n= 10340 | n= 26073 | n= 25207 | <0.001 | 189 | n= 28935 | n= 40082 | n= 11144 | <0.001 | 121 | n= 12193 | n= 44943 | n= 12169 | <0.001 |
| Never, n(%) |  | 6337 (61.29) | 17569 (67.38) | 17378 (68.94) |  |  | 17233 (59.56) | 27911 (69.63) | 8588 (77.06) |  |  | 8259 (67.74) | 29547 (65.74) | 8360 (68.7) |  |
| Former, n(%) |  | 2437 (23.57) | 5391 (20.68) | 4957 (19.67) |  |  | 7134 (24.66) | 7573 (18.89) | 1482 (13.3) |  |  | 2466 (20.22) | 9722 (21.63) | 2183 (17.94) |  |
| Ever, n(%) |  | 1566 (15.15) | 3113 (11.94) | 2872 (11.39) |  |  | 4568 (15.79) | 4598 (11.47) | 1074 (9.64) |  |  | 1468 (12.04) | 5674 (12.62) | 1626 (13.36) |  |
| **Western diet pattern** | 3293 | n= 9783 | n= 24807 | n= 23840 | <0.001 | 4418 | n= 27578 | n= 38194 | n= 10160 | <0.001 | 3965 | n= 11562 | n= 42652 | n= 11247 | <0.001 |
| [-2.93,-0.511], n(%) |  | 2837 (29) | 7266 (29.29) | 6611 (27.73) |  |  | 7315 (26.52) | 10291 (26.94) | 2412 (23.74) |  |  | 3666 (31.71) | 12007 (28.15) | 2976 (26.46) |  |
| (-0.511,0.307], n(%) |  | 2925 (29.9) | 7431 (29.96) | 6849 (28.73) |  |  | 7747 (28.09) | 10280 (26.92) | 2226 (21.91) |  |  | 3386 (29.29) | 12585 (29.51) | 3040 (27.03) |  |
| (0.307,7.78], n(%) |  | 2874 (29.38) | 7121 (28.71) | 7347 (30.82) |  |  | 7602 (27.57) | 10480 (27.44) | 2433 (23.95) |  |  | 3112 (26.92) | 12888 (30.22) | 3238 (28.79) |  |
| Not responding to Q3, n(%) |  | 1147 (11.72) | 2989 (12.05) | 3033 (12.72) |  |  | 4914 (17.82) | 7143 (18.7) | 3089 (30.4) |  |  | 1398 (12.09) | 5172 (12.13) | 1993 (17.72) |  |
| **Physical activity (MET)** | 678 | n= 10251 | n= 25811 | n= 24983 | <0.001 | 893 | n= 28699 | n= 39731 | n= 11027 | <0.001 | 781 | n= 12062 | n= 44513 | n= 12070 | <0.001 |
| (7.57,35.9], n(%) |  | 3158 (30.81) | 8572 (33.21) | 8660 (34.66) |  |  | 8373 (29.18) | 13276 (33.41) | 4869 (44.16) |  |  | 3823 (31.69) | 14210 (31.92) | 4273 (35.4) |  |
| (4.66,7.57], n(%) |  | 3336 (32.54) | 8665 (33.57) | 8423 (33.71) |  |  | 9561 (33.31) | 13528 (34.05) | 3220 (29.2) |  |  | 3955 (32.79) | 15248 (34.26) | 3843 (31.84) |  |
| [0,4.66], n(%) |  | 3757 (36.65) | 8574 (33.22) | 7900 (31.62) |  |  | 10765 (37.51) | 12927 (32.54) | 2938 (26.64) |  |  | 4284 (35.52) | 15055 (33.82) | 3954 (32.76) |  |
| **Height** | 815 | n= 10243 | n= 25738 | n= 24927 | <0.001 | 1079 | n= 28671 | n= 39670 | n= 10930 | <0.001 | 963 | n= 12043 | n= 44439 | n= 11981 | <0.001 |
| [135,160], n(%) |  | 4153 (40.54) | 11663 (45.31) | 11625 (46.64) |  |  | 11691 (40.78) | 18666 (47.05) | 5726 (52.39) |  |  | 5361 (44.52) | 19876 (44.73) | 5816 (48.54) |  |
| (160,164], n(%) |  | 2382 (23.25) | 5805 (22.55) | 5570 (22.35) |  |  | 6569 (22.91) | 8903 (22.44) | 2201 (20.14) |  |  | 2674 (22.2) | 10074 (22.67) | 2490 (20.78) |  |
| (164,190], n(%) |  | 3708 (36.2) | 8270 (32.13) | 7732 (31.02) |  |  | 10411 (36.31) | 12101 (30.5) | 3003 (27.47) |  |  | 4008 (33.28) | 14489 (32.6) | 3675 (30.67) |  |
| **Weight** | 1150 | n= 10181 | n= 25603 | n= 24789 | 0,013 | 1563 | n= 28445 | n= 39477 | n= 10865 | <0.001 | 1352 | n= 11980 | n= 44187 | n= 11907 | <0.001 |
| [29,55], n(%) |  | 3422 (33.61) | 8917 (34.83) | 8328 (33.6) |  |  | 10392 (36.53) | 13350 (33.82) | 3024 (27.83) |  |  | 3679 (30.71) | 15682 (35.49) | 3917 (32.9) |  |
| (55,62], n(%) |  | 3485 (34.23) | 8669 (33.86) | 8402 (33.89) |  |  | 9751 (34.28) | 13237 (33.53) | 3447 (31.73) |  |  | 4069 (33.96) | 14979 (33.9) | 3888 (32.65) |  |
| (62,163], n(%) |  | 3274 (32.16) | 8017 (31.31) | 8059 (32.51) |  |  | 8302 (29.19) | 12890 (32.65) | 4394 (40.44) |  |  | 4232 (35.33) | 13526 (30.61) | 4102 (34.45) |  |
| **Previous ovary cancer (1grade)** | 0 | n= 10369 | n= 26111 | n= 25243 | 0,095 | 0 | n= 29009 | n= 40167 | n= 11174 | 0,044 | 0 | n= 12212 | n= 45011 | n= 12203 | 0,255 |
| No, n(%) |  | 10228 (98.64) | 25793 (98.78) | 24967 (98.91) |  |  | 28648 (98.76) | 39707 (98.85) | 11068 (99.05) |  |  | 12054 (98.71) | 44475 (98.81) | 12073 (98.93) |  |
| Yes, n(%) |  | 141 (1.36) | 318 (1.22) | 276 (1.09) |  |  | 361 (1.24) | 460 (1.15) | 106 (0.95) |  |  | 158 (1.29) | 536 (1.19) | 130 (1.07) |  |
| **Previous breast cancer (1grade)** | 1232 | n= 10156 | n= 25574 | n= 24761 | <0.001 | 1792 | n= 28343 | n= 39337 | n= 10878 | <0.001 | 1440 | n= 11945 | n= 44107 | n= 11934 | <0.001 |
| No, n(%) |  | 4438 (43.7) | 11158 (43.63) | 10675 (43.11) |  |  | 11611 (40.97) | 16985 (43.18) | 4889 (44.94) |  |  | 4844 (40.55) | 18997 (43.07) | 5210 (43.66) |  |
| Yes, n(%) |  | 911 (8.97) | 1977 (7.73) | 1692 (6.83) |  |  | 2273 (8.02) | 2863 (7.28) | 788 (7.24) |  |  | 885 (7.41) | 3280 (7.44) | 897 (7.52) |  |
| Not available, n(%) |  | 4807 (47.33) | 12439 (48.64) | 12394 (50.05) |  |  | 14459 (51.01) | 19489 (49.54) | 5201 (47.81) |  |  | 6216 (52.04) | 21830 (49.49) | 5827 (48.83) |  |
| **MHT use** | 3597 | n= 9727 | n= 24594 | n= 23805 | 0,005 | 8666 | n= 26384 | n= 35817 | n= 9483 | <0.001 | 4000 | n= 11389 | n= 42484 | n= 11553 | <0.001 |
| No, n(%) |  | 8285 (85.18) | 21182 (86.13) | 20632 (86.67) |  |  | 23323 (88.4) | 30945 (86.4) | 8274 (87.25) |  |  | 9484 (83.27) | 36953 (86.98) | 10183 (88.14) |  |
| Yes, n(%) |  | 1075 (11.05) | 2489 (10.12) | 2310 (9.7) |  |  | 2273 (8.62) | 3556 (9.93) | 801 (8.45) |  |  | 1415 (12.42) | 4009 (9.44) | 947 (8.2) |  |
| Undefined, n(%) |  | 367 (3.77) | 923 (3.75) | 863 (3.63) |  |  | 788 (2.99) | 1316 (3.67) | 408 (4.3) |  |  | 490 (4.3) | 1522 (3.58) | 423 (3.66) |  |
| **Number of full term pregnancy** | 3 | n= 10368 | n= 26110 | n= 25242 | <0.001 | 7 | n= 29007 | n= 40165 | n= 11171 | <0.001 | 4 | n= 12212 | n= 45008 | n= 12202 | <0.001 |
| 3+, n(%) |  | 3566 (34.39) | 7520 (28.8) | 6900 (27.34) |  |  | 8039 (27.71) | 11283 (28.09) | 3983 (35.65) |  |  | 3754 (30.74) | 12230 (27.17) | 3374 (27.65) |  |
| 1-2, n(%) |  | 5427 (52.34) | 15748 (60.31) | 15788 (62.55) |  |  | 16181 (55.78) | 25048 (62.36) | 6322 (56.59) |  |  | 6863 (56.2) | 27566 (61.25) | 7287 (59.72) |  |
| 0, n(%) |  | 1375 (13.26) | 2842 (10.88) | 2554 (10.12) |  |  | 4787 (16.5) | 3834 (9.55) | 866 (7.75) |  |  | 1595 (13.06) | 5212 (11.58) | 1541 (12.63) |  |
| **Age at the first childbirth** | 161 | n= 10349 | n= 26035 | n= 25178 | <0.001 | 2636 | n= 28215 | n= 38926 | n= 10573 | <0.001 | 234 | n= 12177 | n= 44877 | n= 12138 | <0.001 |
| [14,23], n(%) |  | 3111 (30.06) | 9031 (34.69) | 9933 (39.45) |  |  | 6088 (21.58) | 16078 (41.3) | 5625 (53.2) |  |  | 4360 (35.81) | 15066 (33.57) | 5016 (41.32) |  |
| (23,26], n(%) |  | 2911 (28.13) | 7209 (27.69) | 6661 (26.46) |  |  | 7604 (26.95) | 10787 (27.71) | 2286 (21.62) |  |  | 3247 (26.67) | 12649 (28.19) | 2799 (23.06) |  |
| (26,59], n(%) |  | 2952 (28.52) | 6953 (26.71) | 6030 (23.95) |  |  | 9736 (34.51) | 8227 (21.13) | 1796 (16.99) |  |  | 2975 (24.43) | 11950 (26.63) | 2782 (22.92) |  |
| No preg, n(%) |  | 1375 (13.29) | 2842 (10.92) | 2554 (10.14) |  |  | 4787 (16.97) | 3834 (9.85) | 866 (8.19) |  |  | 1595 (13.1) | 5212 (11.61) | 1541 (12.7) |  |
| **Combined age and number of pregnancy** | 161 | n= 10349 | n= 26035 | n= 25178 | <0.001 | 2638 | n= 28215 | n= 38926 | n= 10571 | <0.001 | 234 | n= 12177 | n= 44877 | n= 12138 | <0.001 |
| Early first birth and high number of children, n(%) |  | 1556 (15.04) | 3700 (14.21) | 3973 (15.78) |  |  | 2714 (9.62) | 6243 (16.04) | 2650 (25.07) |  |  | 1872 (15.37) | 5971 (13.31) | 1995 (16.44) |  |
| High number of children, n(%) |  | 2001 (19.34) | 3793 (14.57) | 2899 (11.51) |  |  | 5021 (17.8) | 4601 (11.82) | 1108 (10.48) |  |  | 1867 (15.33) | 6206 (13.83) | 1357 (11.18) |  |
| Late first birth and few number of children, n(%) |  | 2158 (20.85) | 5511 (21.17) | 5050 (20.06) |  |  | 7601 (26.94) | 6765 (17.38) | 1418 (13.41) |  |  | 2301 (18.9) | 9658 (21.52) | 2262 (18.64) |  |
| Low number of children, n(%) |  | 3259 (31.49) | 10189 (39.14) | 10702 (42.51) |  |  | 8092 (28.68) | 17483 (44.91) | 4529 (42.84) |  |  | 4542 (37.3) | 17830 (39.73) | 4983 (41.05) |  |
| No pregnancy, n(%) |  | 1375 (13.29) | 2842 (10.92) | 2554 (10.14) |  |  | 4787 (16.97) | 3834 (9.85) | 866 (8.19) |  |  | 1595 (13.1) | 5212 (11.61) | 1541 (12.7) |  |
| **Breastfeeding** | 375 | n= 10310 | n= 25951 | n= 25087 | 0,002 | 8172 | n= 26455 | n= 36235 | n= 9488 | <0.001 | 545 | n= 12111 | n= 44693 | n= 12077 | <0.001 |
| Yes, n(%) |  | 6456 (62.62) | 15740 (60.65) | 15340 (61.15) |  |  | 15910 (60.14) | 21448 (59.19) | 5423 (57.16) |  |  | 7422 (61.28) | 27053 (60.53) | 6759 (55.97) |  |
| No, n(%) |  | 3854 (37.38) | 10211 (39.35) | 9747 (38.85) |  |  | 10545 (39.86) | 14787 (40.81) | 4065 (42.84) |  |  | 4689 (38.72) | 17640 (39.47) | 5318 (44.03) |  |
| **Reproductive lifespan** | 3821 | n= 9742 | n= 24463 | n= 23697 | <0.001 | 5916 | n= 26608 | n= 37543 | n= 10283 | <0.001 | 4392 | n= 11529 | n= 42201 | n= 11304 | <0.001 |
| [27; 41[, n(%) |  | 7743 (79.48) | 19584 (80.06) | 19169 (80.89) |  |  | 21299 (80.05) | 30104 (80.19) | 8427 (81.95) |  |  | 9102 (78.95) | 33933 (80.41) | 9226 (81.62) |  |
| <27, n(%) |  | 124 (1.27) | 342 (1.4) | 385 (1.62) |  |  | 284 (1.07) | 571 (1.52) | 337 (3.28) |  |  | 196 (1.7) | 556 (1.32) | 264 (2.34) |  |
| ≥41, n(%) |  | 1875 (19.25) | 4537 (18.55) | 4143 (17.48) |  |  | 5025 (18.89) | 6868 (18.29) | 1519 (14.77) |  |  | 2231 (19.35) | 7712 (18.27) | 1814 (16.05) |  |
| **Breast cancer screening** | 0 | n= 10369 | n= 26111 | n= 25243 | <0.001 | 0 | n= 29009 | n= 40167 | n= 11174 | <0.001 | 0 | n= 12212 | n= 45011 | n= 12203 | 0,058 |
| Yes, n(%) |  | 7534 (72.66) | 18626 (71.33) | 17772 (70.4) |  |  | 20840 (71.84) | 28402 (70.71) | 7492 (67.05) |  |  | 8739 (71.56) | 32016 (71.13) | 8570 (70.23) |  |
| No, n(%) |  | 2835 (27.34) | 7485 (28.67) | 7471 (29.6) |  |  | 8169 (28.16) | 11765 (29.29) | 3682 (32.95) |  |  | 3473 (28.44) | 12995 (28.87) | 3633 (29.77) |  |
| **Postmenopausal status at baseline** | 2666 | n= 9940 | n= 24955 | n= 24162 | <0.001 | 4240 | n= 27171 | n= 38307 | n= 10632 | <0.001 | 3037 | n= 11776 | n= 43017 | n= 11596 | <0.001 |
| Pre-menopausal, n(%) |  | 5874 (59.09) | 14713 (58.96) | 14702 (60.85) |  |  | 18208 (67.01) | 21780 (56.86) | 4680 (44.02) |  |  | 5933 (50.38) | 27028 (62.83) | 6752 (58.23) |  |
| Post-menopausal, n(%) |  | 4066 (40.91) | 10242 (41.04) | 9460 (39.15) |  |  | 8963 (32.99) | 16527 (43.14) | 5952 (55.98) |  |  | 5843 (49.62) | 15989 (37.17) | 4844 (41.77) |  |
| P-values are estimated with log rank tests. |  |  |  |  |  |  |  |  |  |  |  |  |  |  |  |
| Defined according to the median. |  |  |  |  |  |  |  |  |  |  |  |  |  |  |  |
