# Supplementary material for: The impact of lifecourse socio-economic position and individual social mobility on breast cancer risk
Source: BMC Cancer. 2020 Nov 23;20:1138. doi: 10.1186/s12885-020-07648-w (PMC7684912; doi:10.1186/s12885-020-07648-w)
Supplement: Supplementary file 6 — Additional file 6 Forestplot of the association of the three time point SEP and each covariate used in the fully adjusted model in E3N (N = 83,436). [file 12885_2020_7648_MOESM6_ESM.docx]

Forestplot of the association of the three time point SEP and each covariate used in the fully adjusted model in E3N (N = 83,436).
