# Supplementary material for: The impact of lifecourse socio-economic position and individual social mobility on breast cancer risk
Source: BMC Cancer. 2020 Nov 23;20:1138. doi: 10.1186/s12885-020-07648-w (PMC7684912; doi:10.1186/s12885-020-07648-w)
Supplement: Supplementary file 7 — Additional file 7 Association between life course SEP on the risk of BC using complete cases in E3N [N = 39,122]. [file 12885_2020_7648_MOESM7_ESM.docx]

Association between life course SEP on the risk of BC using complete cases in E3N [N = 39,122].

|  |  | **E3N** | | | | | |
| --- | --- | --- | --- | --- | --- | --- | --- |
|  |  | **A. Father's occupation^a^** | | **B. Education^b^** | | **C. Occupation^a^** | |
|  |  | **Medium** | **Advantaged** | **Middle** | **High** | **Medium** | **Advantaged** |
|  |  | HR [95%CI] | HR [95%CI] | HR [95%CI] | HR [95%CI] | HR [95%CI] | HR [95%CI] |
| **M1** | | 1.03 [0.96; 1.10] | 1.05 [0.96; 1.15] | 1.19 [1.05; 1.35] | 1.30 [1.14; 1.48] | 1.04 [0.95; 1.14] | 1.13 [1.01; 1.26] |
| **M1 + all Health behaviours Anthropometric factors** | | 1.03 [0.96; 1.10] | 1.04 [0.95; 1.14] | 1.17 [1.03; 1.33] | 1.26 [1.11; 1.44] | 1.04 [0.95; 1.14] | 1.12 [1.00; 1.25] |
|  | M1 + age at the first childbirth | 1.02 [0.95; 1.10] | 1.03 [0.94; 1.13] | 1.18 [1.04; 1.34] | 1.22 [1.07; 1.40] | 1.04 [0.95; 1.14] | 1.13 [1.01; 1.26] |
| **M1 + all reproductive factors** | | 1.01 [0.95; 1.09] | 1.01 [0.92; 1.11] | 1.15 [1.01; 1.31] | 1.17 [1.03; 1.34] | 1.03 [0.94; 1.13] | 1.13 [1.01; 1.26] |
| **M2** | | 1.02 [0.95; 1.09] | 1.01 [0.92; 1.10] | 1.15 [1.01; 1.30] | 1.16 [1.01; 1.32] | 1.03 [0.94; 1.13] | 1.13 [1.01; 1.26] |
| M1 is adjusted for age. | |  |  |  |  |  |  |
| ^a^Referent group: "Disadvantaged" | |  |  |  |  |  |  |
| ^b^Referent group: "Low education" | |  |  |  |  |  |  |
| M2 is fully adjusted | |  |  |  |  |  |  |
