# Supplementary material for: The impact of lifecourse socio-economic position and individual social mobility on breast cancer risk
Source: BMC Cancer. 2020 Nov 23;20:1138. doi: 10.1186/s12885-020-07648-w (PMC7684912; doi:10.1186/s12885-020-07648-w)
Supplement: Supplementary file 8 — Additional file 8 Association between life course SEP on the risk of invasive BC only using imputed data in E3N (N = 82,458). [file 12885_2020_7648_MOESM8_ESM.docx]

Association between life course SEP on the risk of invasive BC only using imputed data in E3N (N = 82,458).

|  |  | **E3N** | | | | | |
| --- | --- | --- | --- | --- | --- | --- | --- |
|  |  | **Invasive Breast Cancer [87.6 % of all cases]** | | | | | |
|  |  | **A. Father's occupation^a^** | | **B. Education^b^** | | **C. Occupation^a^** | |
|  |  | **Medium** | **Advantaged** | **Middle** | **High** | **Medium** | **Advantaged** |
|  |  | HR [95%CI] | HR [95%CI] | HR [95%CI] | HR [95%CI] | HR [95%CI] | HR [95%CI] |
| M1 | | 1.03 (0.97; 1.09) | 1.11 (1.02; 1.20) | 1.09 (1.01; 1.17) | 1.20 (1.11; 1.30) | 1.02 (0.95; 1.08) | 1.09 (1.00; 1.18) |
| M1 + all Health behaviours Anthropometric factors | | 1.02 (0.97; 1.09) | 1.09 (1.01; 1.18) | 1.08 (1.00; 1.17) | 1.18 (1.09; 1.28) | 1.01 (0.95; 1.08) | 1.08 (0.99; 1.17) |
| M1 + all reproductive factors | | 1.01 (0.95; 1.07) | 1.06 (0.98; 1.15) | 1.03 (0.95; 1.11) | 1.07 (0.98; 1.16) | 0.98 (0.92; 1.05) | 1.07 (0.98; 1.16) |
| M2 | | 1.01 (0.95; 1.07) | 1.05 (0.97; 1.14) | 1.03 (0.95; 1.11) | 1.06 (0.98; 1.15) | 0.98 (0.92; 1.05) | 1.06 (0.98; 1.15) |
| M1 is adjusted for age. | |  |  |  |  |  |  |
| ^a^Referent group: "Disadvantaged" | |  |  |  |  |  |  |
| ^b^Referent group: "Low education" | |  |  |  |  |  |  |
| M2 is fully adjusted | |  |  |  |  |  |  |
