# Supplementary material for: The impact of lifecourse socio-economic position and individual social mobility on breast cancer risk
Source: BMC Cancer. 2020 Nov 23;20:1138. doi: 10.1186/s12885-020-07648-w (PMC7684912; doi:10.1186/s12885-020-07648-w)
Supplement: Supplementary file 9 — Additional file 9. Characteristics of women with available data from EPIC-Italy according to BC status and by SEP. [file 12885_2020_7648_MOESM9_ESM.docx]

Characteristics of women with available data from EPIC-Italy according to BC status and by SEP.
