# Supplementary material for: The impact of lifecourse socio-economic position and individual social mobility on breast cancer risk
Source: BMC Cancer. 2020 Nov 23;20:1138. doi: 10.1186/s12885-020-07648-w (PMC7684912; doi:10.1186/s12885-020-07648-w)
Supplement: Supplementary file 10 — Additional file 10 Cox proportional hazard regression of BC risk using data from multiple imputation in EPIC-Italy (N = 20,530). [file 12885_2020_7648_MOESM10_ESM.docx]

Cox proportional hazard regression of BC risk using data from multiple imputation in EPIC-Italy (N= 20,530).

|  |  | **A. Father's occupation^a^** | | **B. Education^b^** | | **C. Highest household occupation^a^** | |
| --- | --- | --- | --- | --- | --- | --- | --- |
|  |  | **Medium** | **Advantaged** | **Middle** | **High** | **Medium** | **Advantaged** |
|  |  | **HR [95%CI]** | **HR [95%CI]** | **HR [95%CI]** | **HR [95%CI]** | **HR [95%CI]** | **HR [95%CI]** |
| **M1** | | 1.02 [0.87; 1.19] | 1.06 [0.78; 1.45] | 1.03 [0.88; 1.19] | 1.19 [0.96; 1.47] | 1.03 [0.86; 1.23] | 1.05 [0.86; 1.28] |
| HB-A* | M1 + Alcohol consumption | 1.02 [0.87; 1.19] | 1.06 [0.78; 1.45] | 1.03 [0.89; 1.20] | 1.19 [0.96; 1.48] | 1.03 [0.86; 1.23] | 1.05 [0.86; 1.28] |
|  | M1 + Smoking status | 1.02 [0.87; 1.19] | 1.06 [0.78; 1.45] | 1.03 [0.89; 1.20] | 1.19 [0.96; 1.48] | 1.03 [0.86; 1.23] | 1.05 [0.86; 1.28] |
|  | M1 + Mediterranean diet | 1.02 [0.87; 1.19] | 1.07 [0.78; 1.45] | 1.03 [0.88; 1.19] | 1.19 [0.96; 1.48] | 1.03 [0.86; 1.24] | 1.05 [0.86; 1.28] |
|  | M1 + Physical activity | 1.02 [0.87; 1.20] | 1.05 [0.77; 1.44] | 1.01 [0.87; 1.18] | 1.16 [0.93; 1.45] | 1.01 [0.85; 1.22] | 1.04 [0.86; 1.27] |
|  | M1 + Height | 1.02 [0.87; 1.19] | 1.05 [0.77; 1.43] | 1.02 [0.88; 1.18] | 1.17 [0.95; 1.46] | 1.02 [0.86; 1.22] | 1.04 [0.85; 1.27] |
|  | M1 + Weight | 1.01 [0.87; 1.19] | 1.08 [0.79; 1.48] | 1.05 [0.90; 1.22] | 1.23 [0.99; 1.52] | 1.03 [0.86; 1.24] | 1.06 [0.87; 1.29] |
| **M1 + all Health behaviours Anthropometric factors** | | 1.02 [0.87; 1.20] | 1.08 [0.79; 1.47] | 1.04 [0.89; 1.21] | 1.21 [0.97; 1.52] | 1.02 [0.85; 1.23] | 1.05 [0.86; 1.29] |
| RF* | M1 + age at the first childbirth | 1.03 [0.88; 1.21] | 1.02 [0.75; 1.39] | 0.96 [0.83; 1.12] | 1.05 [0.84; 1.31] | 1.01 [0.85; 1.21] | 1.02 [0.84; 1.25] |
|  | M1 + postmenopausal status | 1.02 [0.87; 1.20] | 1.06 [0.78; 1.44] | 1.02 [0.88; 1.18] | 1.18 [0.95; 1.46] | 1.02 [0.85; 1.22] | 1.04 [0.86; 1.27] |
| **Model 1 + all reproductive factors** | | 1.04 [0.88; 1.21] | 1.02 [0.75; 1.39] | 0.95 [0.82; 1.11] | 1.04 [0.83; 1.30] | 1.00 [0.84; 1.20] | 1.02 [0.83; 1.24] |
| **M2** | | 1.04 [0.89; 1.22] | 1.04 [0.76; 1.42] | 0.97 [0.83; 1.14] | 1.06 [0.85; 1.34] | 1.01 [0.84; 1.22] | 1.03 [0.84; 1.26] |
| M1 is adjusted for age and center | |  |  |  |  |  |  |
| ^a^Referent group: "Disadvantaged" | |  |  |  |  |  |  |
| ^b^Referent group: "Low education" | |  |  |  |  |  |  |
| * HB-A is for health behaviours and anthropometric factors | |  |  |  |  |  |  |
| RF is for reproductive factors | |  |  |  |  |  |  |
| M2 is fully adjusted model | |  |  |  |  |  |  |

Hazard ratio and confidence interval are reported for (A) father’s occupation (B) education and (C) highest household occupation.
