# Supplementary material for: The impact of lifecourse socio-economic position and individual social mobility on breast cancer risk
Source: BMC Cancer. 2020 Nov 23;20:1138. doi: 10.1186/s12885-020-07648-w (PMC7684912; doi:10.1186/s12885-020-07648-w)
Supplement: Supplementary file 11 — Additional file 11. Lifecourse multiple regression analyses of SEP with the future risk of BC in EPIC-Italy using imputed data [N = 20,530]. [file 12885_2020_7648_MOESM11_ESM.docx]

Lifecourse multiple regression analyses of SEP with the future risk of BC in EPIC-Italy using imputed data [N = 20,530].

|  |  |  | **EPIC-Italy** | | | |
| --- | --- | --- | --- | --- | --- | --- |
|  |  |  | **Model A** | **Model B** | **Model C** | **Model D** |
| **Covariates** | **Reference** | **Modality** | HR [95%CI] | HR [95%CI] | HR [95%CI] | HR [95%CI] |
| Father's occupation | Disadvantaged | Medium | 1.01 [0.87; 1.19] | 1.02 [0.87; 1.20] | 1.02 [0.87; 1.20] | 1.04 [0.88; 1.22] |
|  |  | Advantaged | 1.06 [0.78; 1.45] | 1.01 [0.73; 1.39] | 1.00 [0.72; 1.39] | 1.02 [0.74; 1.41] |
| Education | Low | Middle | - | 1.03 [0.88; 1.19] | 1.02 [0.88; 1.19] | 0.97 [0.83; 1.14] |
|  |  | High | - | 1.19 [0.95; 1.49] | 1.18 [0.94; 1.49] | 1.06 [0.83; 1.35] |
| Highest household occupation | Disadvantaged | Medium | - | - | 1.02 [0.85; 1.22] | 1.01 [0.84; 1.21] |
|  |  | Advantaged | - | - | 1.02 [0.83; 1.25] | 1.02 [0.83; 1.26] |
| Model A is adjusted for age, center and father's occupation | |  |  |  |  |  |
| Model B is adjusted for age, center, father's occupation and education | | |  |  |  |  |
| Model C is adjusted for age, center and both SEP | |  |  |  |  |  |
| Model D is adjusted for age, center, both SEP and all covariates [i.e. alcohol consumption, smoking status, physical activity, Mediterranean diet, height, weight, age at first childbirth, menopausal status] | | | | | | |
