# Supplementary material for: The impact of lifecourse socio-economic position and individual social mobility on breast cancer risk
Source: BMC Cancer. 2020 Nov 23;20:1138. doi: 10.1186/s12885-020-07648-w (PMC7684912; doi:10.1186/s12885-020-07648-w)
Supplement: Supplementary file 12 — Additional file 12. Association of social mobility with the risk of BC in EPIC-Italy using imputed data [N = 20,530]. [file 12885_2020_7648_MOESM12_ESM.docx]

Association of social mobility with the risk of BC in EPIC-Italy using imputed data [N = 20,530].

|  |  |  | **EPIC-Italy** | | | |
| --- | --- | --- | --- | --- | --- | --- |
|  |  |  | **Model 1** | **Model 1 + HB-A** | **Model 1 + RF** | **Fully adjusted model** |
| **Covariates** | **Reference** | **Modality** | HR [95%CI] | HR [95%CI] | HR [95%CI] | HR [95%CI] |
| Social mobility | Stable disadvantaged SEP | Downward mobility | 0.99 [0.83; 1.19] | 1.01 [0.84; 1.21] | 1.00 [0.84; 1.20] | 1.01 [0.85; 1.22] |
|  |  | Stable medium SEP | 1.12 [0.84; 1.50] | 1.12 [0.84; 1.50] | 1.12 [0.84; 1.50] | 1.13 [0.85; 1.51] |
|  |  | Upward mobility | 1.00 [0.84; 1.20] | 1.01 [0.84; 1.21] | 0.98 [0.82; 1.18] | 1.00 [0.83; 1.19] |
|  |  | Stable advantaged SEP | 1.32 [0.86; 2.03] | 1.34 [0.87; 2.06] | 1.27 [0.83; 1.94] | 1.30 [0.85; 2.00] |
| Model 1 is adjusted for age, center and social mobility | |  |  |  |  |  |
| Model 1 + HB-A is adjusted for age, center, social mobility, alcohol consumption, smoking status, physical activity, Mediterranean diet, height, weight | | | | |  |  |
| Model 1 + RF is adjusted for age, center, social mobility, age at first childbirth and menopausal status | | | |  |  |  |
| Fully adjusted model is adjusted for age, center, social mobility and all covariates | | |  |  |  |  |
